# Attention deficit hyperactivity and oppositional defiant disorder symptoms in adolescence and risk of substance use disorders—A general population‐based birth cohort study

**DOI:** 10.1111/acps.13588

**Published:** 2023-07-11

**Authors:** Antti Mustonen, Alina Rodriguez, James G. Scott, Miika Vuori, Tuula Hurtig, Anu‐Helmi Halt, Jouko Miettunen, Anni‐Emilia Alakokkare, Solja Niemelä

**Affiliations:** ^1^ Faculty of Medicine and Health Technology Tampere University Tampere Finland; ^2^ Department of Psychiatry Seinäjoki Central Hospital Seinäjoki Finland; ^3^ Research Unit of Population Health University of Oulu Oulu Finland; ^4^ Department of Epidemiology and Biostatistics School of Public Health, Imperial College London UK; ^5^ Centre for Psychiatry and Mental Health Queen Mary University London UK; ^6^ Child Health Research Centre The University of Queensland South Brisbane Queensland Australia; ^7^ Child and Youth Mental Health Children's Health Queensland South Brisbane Queensland Australia; ^8^ Research Center for Child Psychiatry, INVEST Research Flagship University of Turku Turku Finland; ^9^ The Finnish Institute for Health and Welfare Helsinki Finland; ^10^ Research Unit of Clinical Medicine, Psychiatry University of Oulu Oulu Finland; ^11^ Clinic of Child Psychiatry Oulu University Hospital Oulu Finland; ^12^ Medical Research Center Oulu Oulu University Hospital and University of Oulu Oulu Finland; ^13^ Department of Psychiatry Oulu University Hospital Oulu Finland; ^14^ Department of Psychiatry University of Turku Turku Finland; ^15^ Addiction Psychiatry Unit, Department of Psychiatry Turku University Hospital Turku Finland

**Keywords:** ADHD, adolescent, birth cohort, ODD, SWAN

## Abstract

**Background:**

Externalizing symptoms are associated with risk of future substance use disorder (SUD). Few longitudinal studies exist using general population‐based samples which assess the spectrum of attention deficit hyperactivity disorder (ADHD) and oppositional defiant disorder (ODD) symptoms.

**Aims/Objectives:**

We aimed to study the associations between adolescent ADHD symptoms and subsequent SUD and additionally examine whether the risk of SUD is influenced by comorbid oppositional defiant disorder (ODD) symptoms.

**Methods:**

The Northern Finland Birth Cohort 1986 was linked to nationwide health care register data for incident SUD diagnoses until age 33 years (*n* = 6278, 49.5% male). ADHD/ODD‐case status at age 16 years was defined using parent‐rated ADHD indicated by Strengths and Weaknesses of ADHD symptoms and Normal Behaviors (SWAN) questionnaire with 95% percentile cut‐off. To assess the impact of ODD comorbidity on SUD risk, participants were categorized into four groups based on their ADHD/ODD case status. Cox‐regression analysis with hazard ratios (HRs) and 95% confidence intervals (CIs) were used to study associations between adolescent ADHD/ODD case statuses and subsequent SUD.

**Results:**

In all, 552 participants (8.8%) presented with ADHD case status at the age of 16 years, and 154/6278 (2.5%) were diagnosed with SUD during the follow‐up. ADHD case status was associated with SUD during the follow‐up (HR = 3.84, 95% CI 2.69–5.50). After adjustments for sex, family structure, and parental psychiatric disorder and early substance use the association with ADHD case status and SUD remained statistically significant (HR = 2.60, 95% CI 1.70–3.98). The risk of SUD remained elevated in individuals with ADHD case status irrespective of ODD symptoms.

**Conclusions:**

ADHD in adolescence was associated with incident SUD in those with and without symptoms of ODD. The association of ADHD and SUD persisted even after adjustment for a wide range of potential confounds. This emphasizes the need to identify preventative strategies for adolescents with ADHD so as to improve health outcomes.

**Significant Outcomes**

- ADHD case status in adolescence was associated with risk of incident SUD.
- This association was independent of multiple confounders such as adolescent substance use and sex.
- The association of ADHD case status and SUD persisted in individuals with and without symptoms of ODD.

**Limitations**

- Only 3.1% of the sample was diagnosed with SUD which is likely to be underestimate.
- We used parent‐rated ADHD symptom data with cut‐offs to indicate ADHD case status which does not constitute a clinical diagnosis of ADHD.
- Possible role of childhood or familial adversity could not be accounted for.

## 1 INTRODUCTION

Attention Deficit Hyperactivity Disorder (ADHD) is a neurodevelopmental disorder featuring symptoms of inattention, hyperactivity, and impulsivity with onset prior to age of 12.^1^ The community prevalence of ADHD is 6%–7% in youths^2^ and some individuals with ADHD continue to display fluctuating levels of ADHD symptoms and impairments in social functioning in young adulthood.^3^

Past research in longitudinal samples consistently report associations between childhood/adolescent ADHD and early use of licit or illicit substances^4^, ^5^, ^6^ and substance use disorders (SUDs)^4^, ^7^, ^8^, ^9^, ^10^, ^11^, ^12^, ^13^, ^14^ substantiated by meta‐analyses.^15^, ^16^, ^17^, ^18^ However, these data are typically based on clinically diagnosed samples or are relatively small (except for^12^, ^13^). There are very few community‐based studies that have assessed the full spectrum of ADHD symptoms and include participants who have not been clinically diagnosed but have nonetheless elevated symptoms. This is important as ADHD symptoms are associated with significant impairment even at an undiagnosed or sub‐threshold level.^19^, ^20^ Thus, there remains a gap in knowledge concerning persons with elevated ADHD symptoms but who may not have been included in clinical registers.

Whether comorbidity with disruptive behavior disorders (DBD) including conduct disorder (CD) / oppositional defiant disorder (ODD) may account for or have an impact on substance use trajectories with ADHD, remains a subject for debate. To our knowledge, two meta‐analyses have addressed this issue with inconsistent findings. The meta‐analysis by Serra‐Pinheiro et al.^21^ concluded that ADHD alone was not sufficient to increase the risk of illicit substance use/SUD beyond the effect of CD/ODD.^21^ However, even with the combined outcome of both illicit substance use and SUD, the authors acknowledged there were power issues that limited interpretation of their findings. In contrast, the more recent meta‐analysis by Groenman et al.^17^ reported that comorbid ODD/CD with ADHD did not influence the association with SUD.^17^ However, the studies included in the meta‐analysis typically focused on the influence of comorbid CD with ADHD and the evidence base for the effect of ODD is weak. Past research, which is limited to clinical samples, suggests that comorbid ODD with ADHD is not associated with an increased risk for developing SUD.^9^, ^22^ However, the association remains unexamined in community samples, which are less affected by selection bias than clinical samples (e.g., access to care, severity of symptoms, social‐economic factors). Thus, there is a need for large studies to elucidate whether comorbid ODD with ADHD influences the risk of SUD further.

Furthermore, there are only few studies^8^, ^23^ that have been able examine the natural course of ADHD and SUD in an unselected way, for example, by using birth cohort sample. Using this method, all consenting participants from the community are assessed for ADHD and ODD symptoms. Northern Finland Birth Cohort 1986 (NFBC 1986) is a large prospective general population birth cohort,^24^ where individual data for the cohort members are linked to several nationwide registers for all lifetime psychiatric diagnoses. Data used in this study gives opportunity to consider a number of potential confounders such as sex, family structure, parental psychiatric disorders and adolescent substance use when studying the complex associations between adolescent ADHD/ODD and subsequent SUD. Furthermore, NFBC 1986 (over 6000 at follow‐up) is large compared to the previous Dunedin,^23^ and Christchurch Health and Development Studies^8^ that include up to 1200 individuals at enrolment. The substantially larger sample size of this dataset provides more certainty and power to the analysis.

In this sample ADHD / ODD are defined as probable case statuses based on the parent‐rated The Strengths and Weaknesses of ADHD symptoms and Normal Behaviors (SWAN) questionnaire. Using this birth cohort data our aim was to investigate: (1) whether ADHD case status in adolescence is associated with clinical SUD diagnosis up until age 33 years and (2) whether ODD influences the association between ADHD case status and SUD diagnosis.

## 2 MATERIAL AND METHODS

**FIGURE 1:**
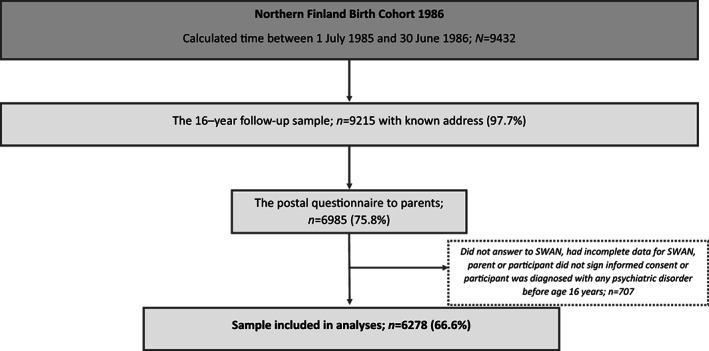
Flowchart of the current study from the Northern Finland Birth Cohort 1986.

NFBC 1986 is an ongoing multidisciplinary birth cohort study comprising 99% of all live‐born children (*n* = 9432) with an expected date of birth between July 1, 1985 and June 30, 1986, from the two northernmost provinces in Finland.^25^ Parents and offspring have been followed‐up in regular intervals including clinical studies at child ages 7–8 years and 15–16 years old. This study concerns the data collected in 2001–2002 when study members were aged 15–16 years. The data collection for the adolescent follow‐up entailed participants and their parents with known addresses. They were sent self‐report questionnaires in separate envelopes (*n* = 9215). Adolescents answered questions concerning their physical health and psychosocial wellbeing (*n* = 7344) and substance use for those participating in the clinical study (*n* = 6798) that took place after the initial postal questionnaire. Parents reported family factors and adolescent ADHD symptoms (*n* = 6985). Participants and parents who signed the informed consent form and with data available on ADHD symptoms were included in the analyses. Figure 1 shows the data flow and the final sample available for analysis (*N* = 6278) after exclusion of those who had been diagnosed with any prior psychiatric disorder (ICD‐10: F00‐F99) before the age of 16 to account for baseline psychiatric comorbidity.

NFBC 1986 study is approved by the Ethics committee of the Northern Ostrobothnia Hospital District in Finland with latest version dated on January 15, 2018 (EETTMK 108/2017). The authors assert that all procedures contributing to this work comply with the ethical standards of the relevant national and institutional committees on human experimentation and with the Helsinki Declaration of 1975, as revised in 2008.

### 2.1 Outcome variable: substance use disorder

Participants' data were linked to register‐based substance use disorder (SUD) diagnoses (F1x.x) from age of 16 years until the end of 2018 when participants were aged 33 years. The onset age of each disorder was based on the first record of the diagnosis in registers. SUD diagnoses were obtained from linkage to four nationwide registers providing extensive coverage on diagnosed psychiatric disorders and only minimal attrition. Data on diagnoses were collected from the Care Register for Health Care 2001–2018, the Register of Primary Health Care Visits 2011–2018, the medication reimbursement register of the Social Insurance Institution of Finland 2001–2005 and the disability pensions of the Finnish Center for Pensions 2001–2016. The Care Register contains information on patients discharged from inpatient care, and since 1998 also on specialized outpatient care. The Register of Primary Health Care Visits includes all outpatient primary health care delivered in Finland. These registers are detailed in previous research.^26^, ^27^, ^28^ Information on deaths and times of emigration were obtained from the Population Register data.

### 2.2 Exposure variable: ADHD and ODD symptoms in adolescence reported by parent

The Strengths and Weaknesses of ADHD symptoms and Normal Behaviors (SWAN) questionnaire^29^, ^30^ is a revised version of SNAP‐IV developed by Swanson and his colleagues. SWAN measures problems in attention, hyperactivity/impulsivity, and disruptive behavior.^29^, ^30^ This study used a SWAN version where the items are based on the 18 ADHD symptoms and 8 ODD symptoms described in the DSM‐IV‐TR for example “Gives close attention to detail and avoids careless mistakes.” Parents rate these items on a 7‐point scale anchored to average behavior (i.e., Far Below Average = 3, Below Average = 2, Somewhat Below Average = 1, Average = 0, Somewhat Above Average = −1, Above Average = −2, and Far Above Average = −3) resulting in normally distributed behavioral ratings. Individual SWAN items were used to compute means for a Combined ADHD scale score (C), using all 18 items, an Inattentive score (I) using 9 Inattentive items, and a Hyperactive–Impulsive score (HI) using 9 Hyperactive–Impulsive items and Oppositional defiant disorder scale (ODD) using 8 Oppositional defiant disorder items. The SWAN scores were used to identify probable ADHD “cases” by requiring SWAN scores to exceed the >95th percentile on either C or I or HI scales.^31^ Participants who scored below the 95th percentile on all three ADHD scales were classified as “controls.” We excluded participant data if there were two or more items missing in one of the SWAN scales. If there was only one item missing in any SWAN scale, the missing value was replaced by the mean value of the items of that particular scale for that person. To study the effect of comorbid ODD, we defined ODD case status as exceeding 95th percentile of the symptom distribution in SWAN ODD. The data on ADHD / ODD case statuses were then categorized into four categories (1) Positive ADHD and ODD case statuses, (2) Positive ADHD case status only, (3) Positive ODD case status only, and (4) controls (participants below 95% percentile in each of these categories). For the questionnaire used in this study, see NFBC website: https://www.oulu.fi/nfbc/node/18149.

#### 2.2.1 Covariates

Early risk factors for substance use disorders have been previously described in the literature^32^, ^33^, ^34^; these variables have also been found to associate with ADHD.^35^, ^36^ Therefore, to clarify the association between ADHD during adolescence and SUD in adulthood, we adjust the analyses with the following covariates.

#### 2.2.2 Self‐reported substance use at age 15/16 years

Adolescents reported the frequency of alcohol intoxications during the past year. Participants were asked: “Have you been drunk during the past year? (0, 1–2, 3–5, 6–9, 10–19, 20–39 or 40 times or more).” We dichotomized this variable as ≥10 times (=1) and ≤9 (=0) based on the distribution of data.

To assess lifetime cannabis use until follow‐up at age 16 years, adolescents were asked: “Have you used marihuana or hashish?” with options “never,” “once,” “two to four times,” “five or more times,” or “I use regularly.” These options were pooled as “lifetime cannabis use” yes (=1) or no (=0) to provide sufficient sample size.

Adolescents were also asked “Have you tried or used any of the following substances?—Ecstasy, heroin, cocaine, amphetamine, LSD or other similar intoxicating drugs?” and “Have you ever tried or used any of the following substances?—Sniffing thinner, glue, etc. for intoxication” with options “never,” “once,” “two to four times,” “five or more times,” or “I use regularly.” The data on other illicit substances other than cannabis and use of inhalants were pooled into a binary variable “other lifetime substance use” yes (=1) or no (=0) to provide sufficient sample size.

#### 2.2.3 Family structure

Information on family structure was collected by combining data from parents at birth and from the clinical study in 2001–2002. These data were categorized as “family with two biological parents (=0),” where both biological parents lived together with the participant, and “other (=1),” which consisted of all other family types.

#### 2.2.4 Parental psychiatric disorders

Data on parental psychiatric diagnoses including substance use disorders (F00‐69, F80‐99) were obtained up until 2018 from the national registers, which included: (1) Register of Health Care 1972–2018 (including inpatient care and visits to specialized outpatient health care since 1998); (2) Disability pensions of the Finnish Centre for Pensions (1965–2016); and (3) The Register of Primary Health Care Visits (2011–2018).

### 2.3 Statistical methods

The statistical analyses were performed using SPSS statistical software (IBM SPSS Statistics, version 28; IBM Co., Armonk, New York, USA) and R (R Foundation for Statistical Computing, version 4.2.0; R Core Team., Armonk, Vienna, Austria) packages Epi and survival. Association of between covariates, ADHD case status and incident SUD diagnosis was assessed with chi‐square test. The association ADHD case status and risk of SUD was examined using Cox regression analysis with hazard ratios (HR) and 95% confidence intervals (95% CI). Times at emigration outside the country (*n* = 247) and death (*n* = 60) were used as censoring points. Statistical significance was defined as *p* ≤ 0.05. We used the following models to adjust for potential confounders: Model 1 crude; Model 2: sex, family structure and any parental psychiatric disorder; Model 3: additionally, lifetime cannabis use, other lifetime substance use, frequent alcohol intoxication. Furthermore, we studied risk of SUD in different ADHD/ODD case status categories by using Cox‐regression analysis. The Aalen–Johansen cumulative incidence curves were computed for the Cox‐regression models. Linear regression and multicollinearity diagnostics with variance inflation factor (VIF) scores were used to examine possible correlation between multiple covariates (variables in Model 3).

We considered known attrition due to non‐participation in this sample.^37^ Fewer males (64% vs. 71%; *p* < 0.001), individuals living in urban areas (66% vs. 71%, *p* < 0.001) and individuals with parental psychiatric disorder (58% vs. 69%, *p* < 0.001) were less likely to participate in the 15–16‐year follow‐up study.^37^ We addressed this attrition by weighing our main analyses by sex, parental psychiatric disorder, and urbanicity by using inverse probability weighting^38^ and analyzed these data with logistic regression analysis and odds ratios (OR) (Figure S1).

To assess the stability of our results, we conducted a set of sensitivity analyses. First, we used in Cox‐regression analysis with hazard ratios (HR) and 95% CI in Model 3 without restricting the sample for participants psychiatric disorder prior age 16 years (Figure S1). Second, we studied the association of ADHD case status and SUD in Models 1–3 using SWAN 90th percentile as cut‐off (Figure S1), which reflects symptoms at a sub‐threshold level.

## 3 RESULTS

The final sample for analysis included 6278 (49.5% male) individuals as shown in Figure 1. Of these individuals 552 (8.8%) presented with ADHD case status at the age of 16 years. During the follow‐up from 2001 to 2018, that is, from age 16 to 33 years, 154/6278 (2.5%) individuals were diagnosed with SUD.

Those reaching case status for ADHD at 16 years were more likely to be male, come from families where both biological parents did not live with the participant, and were also more likely to live in families with parental psychiatric disorder. Furthermore, they were also more likely to report lifetime substance use and frequent alcohol intoxication past year and to be diagnosed with incident SUD compared to those who did not (Table 1).

**TABLE 1:** Association of covariates and ADHD case status and incident substance use disorder (SUD) in Northern Finland Birth Cohort 1986.

	Total *n*	*n* = 6278			ADHD case status					Incident substance use disorder				
					No ADHD case status *n* = 5726		ADHD case status *n =* 552		*p*‐Value	No incident SUD *n* = 6124		Incident SUD *n* = 154		*p*‐Value
		*n*	%		*n*	%	*n*	%		*n*	%	*n*	%	
*Sex*	6278													
Male		3106	49.5		2769	48.4	337	61.1	<0.001	3002	49.0	104	67.5	<0.001
Female		3172	51.5		2957	51.6	215	38.9		3122	51.0	50	32.5	
*Family structure*	6148													
Other		1354	22.0		1179	21.0	175	33.1	<0.001	1294	21.6	60	40.8	<0.001
Family with two biological parents		4794	78.0		4441	79.0	353	66.9		4707	78.4	87	59.2	
*Lifetime cannabis use*	5380													
No		5089	94.6		4701	95.1	388	88.6	<0.001	4992	94.9	97	80.2	<0.001
Yes		291	5.4		241	4.9	50	11.4		267	5.1	24	19.8	
*Other lifetime substance use*	5406													
No		5222	96.6		4818	97.0	404	91.8	<0.001	5122	96.9	100	82.6	<0.001
Yes		184	3.4		148	3.0	36	8.2		163	3.1	21	17.4	
*Alcohol intoxication 10 ≥ times past year*	5293													
No		4336	81.9		4038	83.1	298	68.8	<0.001	4266	82.5	70	58.8	<0.001
Yes		957	18.1		822	16.9	135	31.2		908	17.6	49	41.2	
*Parental psychiatric disorder*	6278													
No		3982	63.4		3672	64.1	310	56.2	<0.001	3918	64.0	64	41.6	<0.001
Yes		2296	36.6		2054	35.9	242	43.8		2206	36.0	90	58.4	
*Incident SUD*	6278													
No		6124	97.5		5613	98.0	511	92.6	<0.001	–	–	–	–	–
Yes		154	2.5		113	2.0	41	7.4		–	–	–	–	
*ADHD case status*	6278													
No		5726	91.2		–	–	–	–	–	5613	91.7	113	73.4	<0.001
Yes		552	8.8		–	–	–	–		511	8.3	41	26.6	

**FIGURE 2:**
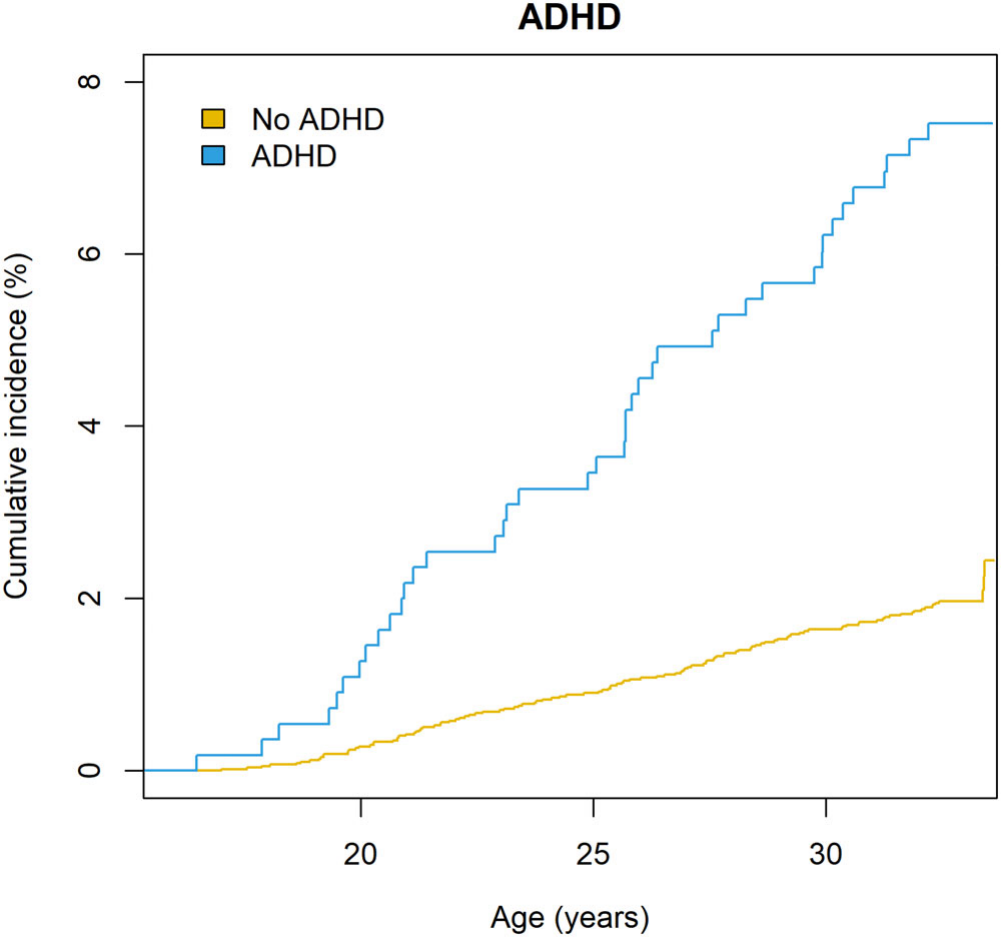
The Aalen–Johansen cumulative incidence curve for the association between ADHD case status and substance use disorder.

Cumulative incidence curve for the association between ADHD case status and SUD is presented in Figure 2. In the crude analyses (Model 1) ADHD case status was associated with SUD during the follow‐up at a statistically significant level (HR = 3.84, 95% CI 2.69–5.50). After adjustments for sex, family structure, and parental psychiatric disorder (Model 2), the association of ADHD case status and SUD attenuated but remained statistically significant (HR = 2.97, 95% CI 2.04–4.32). The association attenuated further but remained still statistically significant (HR = 2.60, 95% CI 1.70–3.98) after adjustment for frequent alcohol intoxication past year and lifetime cannabis and other substance use until adolescence (Model 3, see Table 2). In these final models, frequent alcohol intoxication, lifetime cannabis use and other substance use, family structure, parental psychiatric disorder, and male sex were also associated with SUD (Table S1). The results of our primary analyses aligned with our sensitivity analysis that were not restricted for baseline psychiatric diagnoses or use of the SWAN 90% cutoff when defining ADHD case status (Figure S1). Furthermore, all the statistically significant ORs in unweighted analyses were also statistically significant in the weighted analyses, and the strength of the associations were of similar magnitude (see Figure S1). Multicollinearity was not seen (Model 3, all VIFs <1.2).

**TABLE 2:** Association of ADHD case status and risk of substance use disorder (SUD).

	Sample size	Incident SUD cases	HR	95% CI
Model 1	6278	154	**3.84**	**2.69–5.50**
Model 2	6138	147	**2.97**	**2.04–4.32**
Model 3	5151	115	**2.60**	**1.70–3.98**

*Note*: Model 1: crude, Model 2: sex. family structure, parental psychiatric disorder, Model 3: Model 2, lifetime cannabis use, other lifetime substance use, frequent alcohol intoxication past year. Statistically significant results in bold.

Abbreviations: 95% CI, 95% confidence intervals; ADHD, Attention‐deficit/hyperactivity disorder; HR, hazard ratio; SUD, substance use disorder.

**FIGURE 3:**
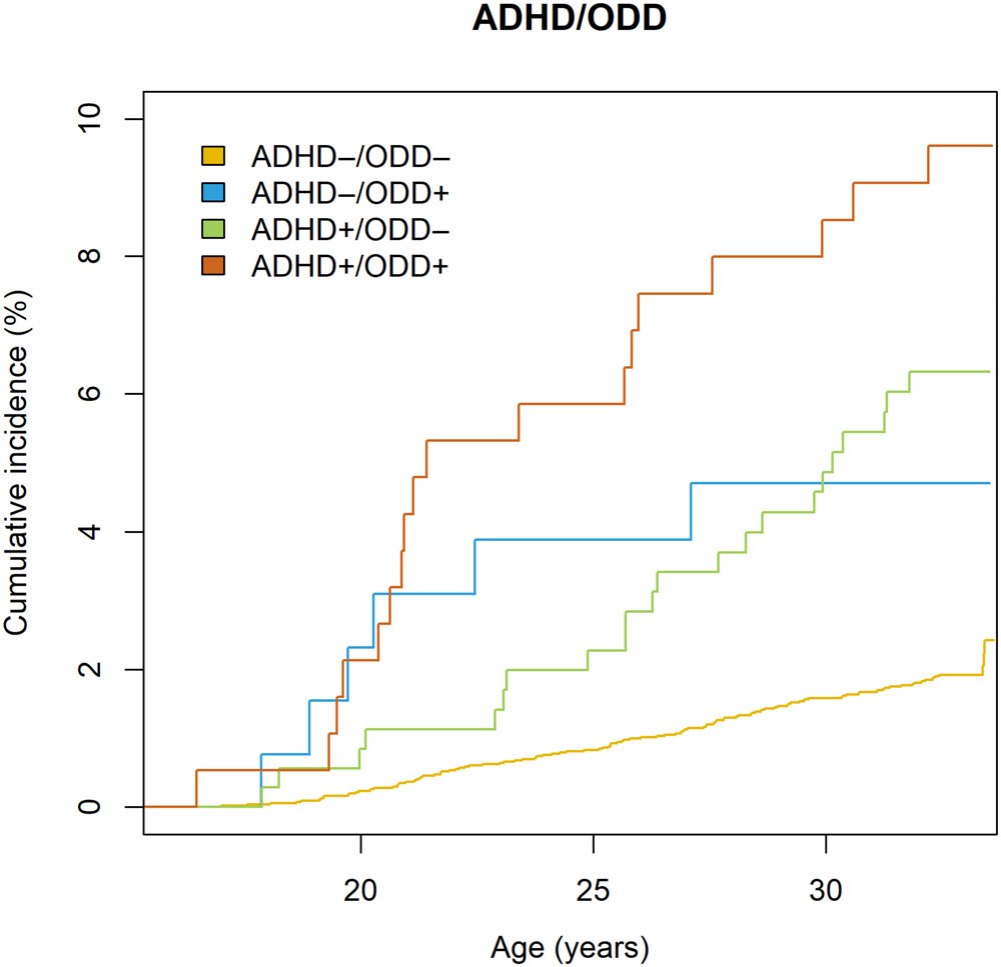
The Aalen–Johansen cumulative incidence curve for the association between ADHD/ODD case status categories and substance use disorder.

Complete SWAN ADHD and ODD data were available for 6165 individuals of which 152 (2.5%) were diagnosed with SUD during the follow‐up. There were 188 individuals (3.0% of the sample) individuals with ADHD+/ODD+ case status, 355 individuals (5.7% of the sample) with ADHD+/ODD‐ case status, 130 individuals (2.1% of the sample) with ADHD−/ODD+ case status and 5492 individuals (87.5% of the sample) with ADHD−/ODD‐ case status (see Table 3). Individuals with ADHD+/ODD+ case status were more likely be diagnosed with SUD compared to controls (HR = 5.13, 95% CI 3.11–8.45) as were individuals with ADHD+/ODD‐ case status (HR = 3.26, 95% CI 2.06–5.16) and ADHD−/ODD+ case status (HR = 2.54, 95% CI 1.11–5.77). For cumulative incidence curve, see Figure 3.

**TABLE 3:** Association of ADHD/ODD case status categories and risk of substance use disorder.

	Sample size	HR	95% CI
ADHD−/ODD−	5492	Ref.	
ADHD−/ODD+	130	**2.54**	**1.14–5.77**
ADHD+/ODD‐	355	**3.26**	**2.06–5.16**
ADHD+/ODD+	188	**5.13**	**3.11–8.45**

*Note*: Statistically significant results in bold.

Abbreviations: 95% CI, 95% confidence intervals; ADHD, Attention‐deficit/hyperactivity disorder; HR, hazard ratio; ODD, Oppositional defiant disorder; SUD, substance use disorder.

## 4 DISCUSSION

Using general population‐based sample of Northern Finland Birth Cohort 1986, we report an association of parent‐rated ADHD based on probable SWAN caseness and risk of SUD diagnosed in clinical practice until 33 years of age. This association was independent of sex, family structure through early life, parental psychiatric disorders, and adolescent substance use. Furthermore, this association with SUD was seen in individuals reaching ADHD case status irrespective of ODD symptoms.

This study adds new knowledge pertaining to a general population sample and points to ADHD and ODD at the probable case level in adolescence to be associated with SUD up until the age of 33 years. Furthermore, our sensitivity analyses suggested that individuals with ADHD‐like traits at the low and high extreme end of the distribution are at risk of SUD. Use of register data for all cohort participants were available with the benefit of long‐term follow‐up without confounding with ADHD/ODD case status. Our findings align with the previous reports of positive associations of ADHD and SUD in clinical studies,^9^, ^10^ register and twin samples^4^, ^7^, ^12^, ^13^ and bolster previous cohort findings.^8^, ^11^

This study was able to assess individual and family level confounding due to sex, parental psychiatric disorders, family structure and adolescent substance use. These are all prominent risk factors for SUD that also associate with ADHD, and so, could potentially explain away the association between ADHD and SUD. In our analysis, the effect sizes attenuated by increasing adjustments as expected. Yet, ADHD case status remained at more than twofold risk for incident SUD. This suggests that association of ADHD and SUD was not explained by familial or individual confounding including adolescent substance use. The latter is particularly important as it is known that ADHD increases the risk of early use of licit and illicit substances^4^, ^5^, ^6^ and this in turn is a strong predictor for future SUD.^39^ Thus, these findings suggest ADHD may confer to the risk of SUD even without the influence of adolescent substance use and other prominent risk factors.

We report that cohort members with both ADHD/ODD case statuses had the greatest point estimates and cumulative incidences of SUD during the follow‐up. Yet, ADHD and ODD case statuses contributed to the risk of future SUD also individually with confidence intervals overlapping between these three groups. Thus, our findings suggest that individuals with ADHD are at risk of SUD irrespective of comorbid ODD symptoms. In this respect our findings are consistent with the most recent meta‐analysis that the association of ADHD and SUD was not dependent on CD / ODD.^17^ However, the novelty of this study is that we were able to focus on comorbidity with ODD using data from more than 6000 participants in contrast to the previous individual studies that are mostly based on smaller clinical samples that have typically focused on CD.

Our results have clinical implications. Based on our findings and literature to date ADHD increases the risk of SUD. Past research suggests that this comorbidity associates with further risks for general medical conditions, mortality, and disability burden.^40^, ^41^, ^42^, ^43^ There is evidence that stimulant treatment for ADHD mitigates the development of SUD especially when treatment is initiated early and for longer durations.^44^ Further, children who had been diagnosed with ADHD during childhood are shown to have lower risks for SUD in early adulthood than those who were diagnosed with ADHD during adolescence or early adulthood (5.2% vs. 9.4% vs. 14.3%) suggesting delayed diagnoses of ADHD may increase the risk for SUD due to delayed treatment onset.^45^ Backed by this evidence, our results suggest that it is vital to identify and treat ADHD to prevent or mitigate the progression of substance use to a clinical SUD. Our results further suggest that emphasis should be also placed on individuals with externalizing comorbidity such as ODD, yet the risk of SUD remains elevated in ADHD and ODD beyond their comorbidity.

The NFBC 1986 study is among the largest birth cohort studies in the world with considerable follow‐up that allows for robust examination of prospective associations into adulthood. This study was able to examine the associations of ADHD/ODD symptoms and SUD by combining questionnaire data to national registers for ICD‐10 diagnosis codes with a prospective general population‐based design with 18 years of follow‐up. Furthermore, the extensive dataset allowed us to evaluate the association while considering several potential confounders.

There are also limitations. Information on substance use during adolescence was collected retrospectively via self‐report questionnaire at age 15–16 years, which may potentially have been underreported. Although SWAN cut‐offs are used as probable ADHD diagnosis, it does not constitute a full psychiatric clinical assessment. Nonetheless, this definition sheds light on the impact of elevated ADHD symptoms regardless of clinical status. This study identified 8.8% of the sample with ADHD case status which is somewhat higher than the community prevalence of clinical ADHD (5.9%–7.2%) in a previous meta‐analysis.^2^ However, studies with no requirement of impairment tend to have 2.3% higher prevalence estimates that those with requirement.^46^ As the ADHD−/ODD+ category was small with only 130 individuals the effect size reported in this paper may be an underestimate. We did not have data on CD for the cohort and thus we were not able examine whether comorbid CD in ADHD influences SUD risk further. Despite substantial sample size, it was not possible to stratify our analyses by sex due to power issues. Furthermore, 3.1% of the whole study population was diagnosed with SUD which is likely to be an underestimate.^47^ Possible reasons are underdiagnosis and the fact that part of the substance use services are administered in social services which does not contribute data to the registers used in this study. Lastly, possible role of childhood or familial adversity could not be accounted for. To conclude, ADHD case status in adolescence associated with incident SUD independent of sex, demographic factors, parental psychiatric disorder, and early substance use in the general population‐based Northern Finland Birth Cohort with 18 years of follow‐up. Moreover, the risk of SUD remained elevated in individuals reaching ADHD case status whether or not they had ODD symptoms. ADHD is a serious neurodevelopmental disorder requiring optimal management to prevent future SUD and other serious health problems.

## AUTHOR CONTRIBUTIONS

Antti Mustonen, Jouko Miettunen, and Solja Niemelä developed conception and design of the work. Antti Mustonen and Anni‐Emilia Alakokkare performed data analysis and interpretation. Antti Mustonen, Solja Niemelä, Jouko Miettunen, Miika Vuori, Alina Rodriguez, Tuula Hurtig, Anu‐Helmi Halt, and James G. Scott wrote the first manuscript draft. Antti Mustonen, Solja Niemelä, Jouko Miettunen, Alina Rodriguez, Miika Vuori, Tuula Hurtig, Anu‐Helmi Halt, James G. Scott, and Anni‐Emilia Alakokkare supervised conception and design of the work and provided critical revision of the article. All authors contributed approval of the final version of the manuscript.

## FUNDING INFORMATION

NFBC1986 has received funding from EU QLG1‐CT‐2000‐01643 (EUROBLCS) Grant no. E51560, NorFA Grant no. 731, 20056, 30167, USA/NIH 2000G DF682 Grant no. 50945. For this study, Antti Mustonen has received funding from Juho Vainio Foundation, Emil Aaltonen Foundation, The Hospital District of South Ostrobothnia, and The Finnish Foundation for Alcohol Studies. Anu‐Helmi Halt and Tuula Hurtig have received funding from Terttu foundation. Solja Niemelä has received funding from Juho Vainio foundation, Sohlberg foundation. James G. Scott is supported by a National Health and Medical Research Council Practitioner Fellowship (APP1105807). Jouko Miettunen has received funding from Juho Vainio Foundation and from the Yrjö Jahnsson Foundation. Miika Vuori reports no potential conflicts of interest.

## CONFLICT OF INTEREST STATEMENT

Solja Niemelä has received speaker fees (Shire‐Takeda), and travel fees (Shire‐ Takeda). Anu‐Helmi Halt has received travel fees (Lundbeck). Others report no potential conflicts of interest.

### PEER REVIEW

The peer review history for this article is available at https://www.webofscience.com/api/gateway/wos/peer-review/10.1111/acps.13588.

## Supporting information


**Figure S1:** Association of ADHD case status and risk of SUD in sensitivity analyses.

**Table S1:** Association of ADHD case status, covariates, and risk of substance use disorder (SUD) in Northern Finland Birth Cohort 1986.

## Data Availability

NFBC data are available from the University of Oulu, Infrastructure for Population Studies. Permission to use the data can be applied for research purposes via electronic material request portal. In the use of data, we follow the EU general data protection regulation (679/2016) and Finnish Data Protection Act. The use of personal data is based on cohort participant's written informed consent at his/her latest follow‐up study, which may cause limitations to its use. Please, contact NFBC project center (NFBCprojectcenter(at)oulu.fi) and visit the cohort website for more information.
