# Supplementary figures and images for: Attention deficit hyperactivity and oppositional defiant disorder symptoms in adolescence and risk of substance use disorders—A general population‐based birth cohort study
Source: Acta Psychiatr Scand. 2023 Jul 11;148(3):277–87. doi: 10.1111/acps.13588 (PMC10953420; doi:10.1111/acps.13588)

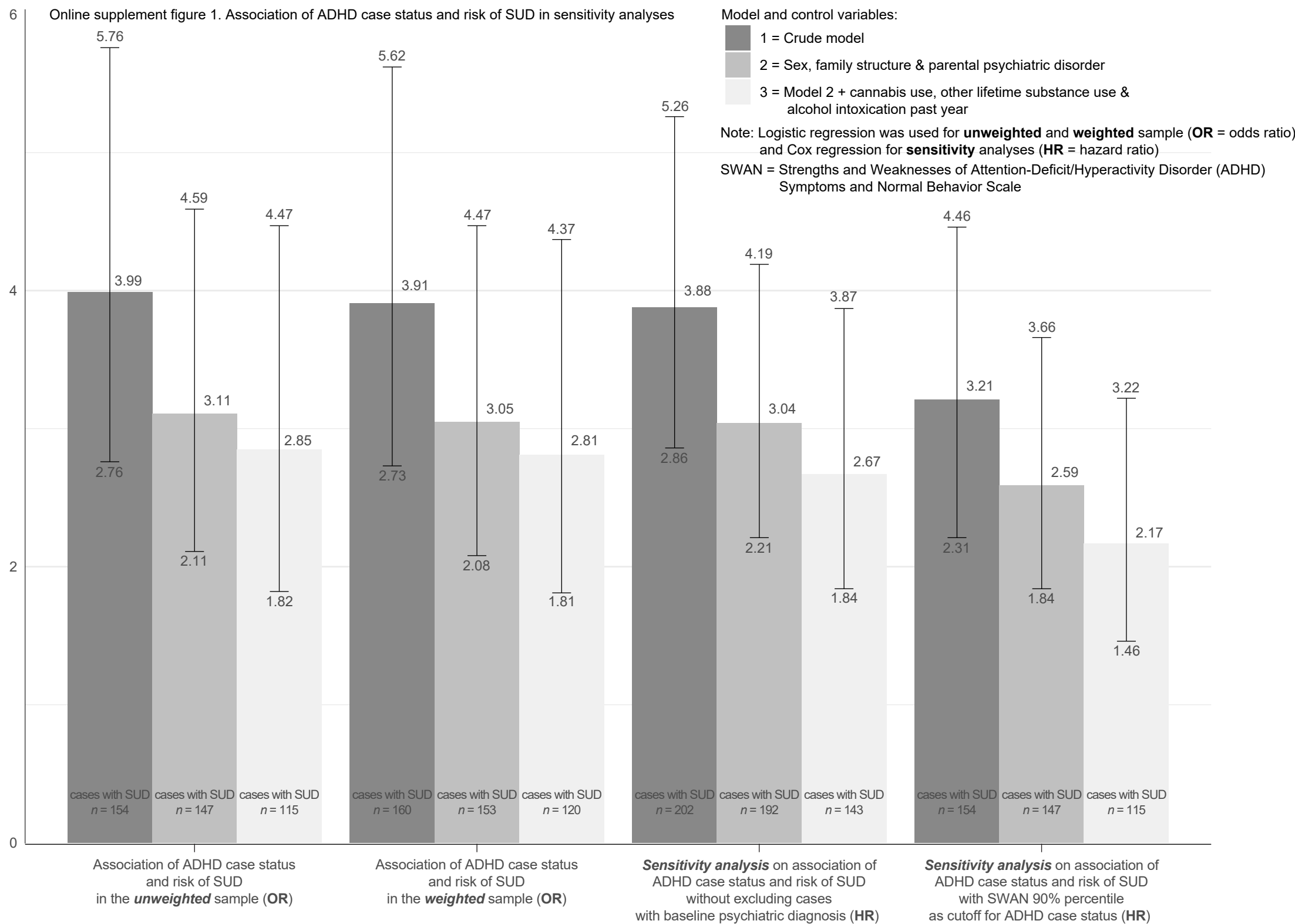

Supplement: Supplementary file 1 — Figure S1: Association of ADHD case status and risk of SUD in sensitivity analyses. [file ACPS-148-277-s002.pdf]
