# Supplementary material for: Attention deficit hyperactivity and oppositional defiant disorder symptoms in adolescence and risk of substance use disorders—A general population‐based birth cohort study
Source: Acta Psychiatr Scand. 2023 Jul 11;148(3):277–87. doi: 10.1111/acps.13588 (PMC10953420; doi:10.1111/acps.13588)
Supplement: Supplementary file 2 — Table S1: Association of ADHD case status, covariates, and risk of substance use disorder (SUD) in Northern Finland Birth Cohort 1986. [file ACPS-148-277-s001.docx]

| **Online supplement table 1. Association of ADHD case status, covariates, and risk of substance use disorder (SUD) in Northern Finland Birth Cohort 1986.** | | | |
| --- | --- | --- | --- |
| **Covariates in Model 3** | HR | 95% CI |  |
| ADHD case status | **2.60** | **1.70-3.98** |  |
| Sex* | **0.44** | **0.30–0.65** |  |
| Parental psychiatric disorder | **2.51** | **1.71–3.69** |  |
| Family structure | **1.77** | **1.21–2.61** |  |
| Frequent alcohol intoxications past year | **1.81** | **1.18–2.77** |  |
| Lifetime cannabis use | **2.13** | **1.26–3.58** |  |
| Other lifetime substance use | **2.79** | **1.64–4.76** |  |
| Statistically significant results in bold. Abbreviations: 95% CI = 95% confidence intervals, HR = hazard ratio. *Male sex reference | | |  |
|  |  |  |  |
